# My Baby, My Move+: feasibility of a community prenatal wellbeing intervention

**DOI:** 10.1186/s40814-023-01368-1

**Published:** 2023-07-28

**Authors:** Jenn A. Leiferman, Rachael Lacy, Jessica Walls, Charlotte V. Farewell, Mary K. Dinger, Danielle Symons Downs, Sarah S. Farrabi, Jennifer L. Huberty, James F. Paulson

**Affiliations:** 1 Colorado School of Public Health, University of Colorado | Anschutz Medical Campus, 13001 East 17th Place, Aurora, CO 80045 USA; 2 Department of Kinesiology, College of Health and Human Development, The Pennsylvania State University, State College, PA USA; 3 Department of Obstetrics and Gynecology, College of Medicine, The Pennsylvania State University, 266 Recreation Building University Park, State College, PA 16802 USA; 4 Goldfarb School of Nursing at Barnes-Jewish College, 4483 Duncan Ave, St. Louis, MO 63110 USA; 5 Center for Human Nutrition, Washington University School of Medicine, 660 S. Euclid, St. Louis, MO 63110 USA; 6 College of Health Solutions, Arizona State University, 500 North 3rd Street, Phoenix, AZ 85004 USA; 7 Department of Psychology, Old Dominion University, 5115 Terminal Blvd, Norfolk, VA 23529 USA

**Keywords:** Prenatal, Perinatal, Pregnancy, Feasibility, Wellbeing, Peer intervention

## Abstract

**Background:**

Excessive gestational weight gain (EGWG), insufficient prenatal physical activity and sleep, and poor psychological wellbeing independently increase risks for adverse maternal and infant outcomes. A novel approach to mitigate these risks is utilizing peer support in a community-based prenatal intervention. This study assessed the feasibility (acceptability, demand, implementation, and practicality) of a remotely delivered prenatal physical activity intervention called My Baby, My Move + (MBMM +) that aims to increase prenatal physical activity, enhance mood and sleep hygiene, and reduce EGWG.

**Methods:**

Participants were recruited through community organizations, local clinics, and social media platforms in the Fall of 2020 and Spring of 2021. Eligible pregnant women were randomized to either the MBMM + intervention or the control group. Each group met over Zoom for 16 sessions (twice weekly for 60 min over 8 weeks) to learn either behavioral change and wellbeing knowledge and skills (MBMM +) or knowledge and skills related to parenting (control group). Multiple methods of evaluation to better understand the feasibility of the intervention were conducted.

**Results:**

A total of 49 women (25 MBMM + intervention, 24 control) completed both pre- and post-survey assessments and were included in the analyses. A subsample of 19 (39%) intervention participants completed a combination of semi-structured interviews/surveys to assess acceptability, demand, implementation, and practicality. Participants expressed positive feedback regarding acceptability (satisfaction and intent to continue use) and were *extremely likely* or *likely* to recommend the program to a friend (demand). Implementation metrics were assessed by observation and feedback forms completed by peer leaders and demonstrated high-quality control. Findings suggest that the intervention was practical due to remote sessions and cost-effectiveness.

**Conclusion:**

The MBMM + intervention was deemed to be a feasible intervention with high acceptability, demand, implementation, and practicality. These findings can be used to inform the scalability of the intervention and implementation of a larger efficacy trial.

**Trial registration:**

19–1366, initial date is on January 23, 2020.

## Key messages regarding feasibility

- The remote delivery platform of MBMM + may influence components of feasibility (especially practicality).
- MBMM + showed high feasibility across acceptability, demand, implementation, and practicality.
- Feasibility findings from this study will inform the testing of MBMM + in a larger efficacy trial to better understand its impact on indicators of maternal health.

## Background

The USA has the highest rate of maternal mortality among high-income countries [1], and this rate is continuing to increase [2]. Contributors to maternal mortality include preventable adverse pregnancy and birth outcomes that may stem from excessive gestational weight gain (EGWG) [3, 4]. Approximately half of pregnant women in the USA gain more than the recommended weight during pregnancy based on the Institute of Medicine (IOM) guidelines [5]. EGWG increases the risk of complications throughout pregnancy such as gestational diabetes [6, 7], pre-eclampsia [8], and adverse birth outcomes including preterm birth [9] and the need for cesarean birth [10] and medical induction interventions [11]. EGWG also increases the risk of excessive maternal weight retention, obesity, and associated chronic diseases during the postpartum period and beyond [8, 12–15].

Traditional interventions aimed to reduce EGWG have focused primarily on nutrition/dietary changes [16–18]. However, results related to sustaining these behavior changes into the postpartum period are conflicting [13, 17, 19–22]. This may be due in part to the variation in health care provider recommendations, as well as understanding and compliance with recommendations [23, 24]. A recent review highlights the need to identify specific modifiable factors related to gestational weight gain to design more effective EGWG interventions [25]. For example, interventions that focus on physical activity have shown to be effective in the prevention of EGWG [19, 26, 27] and when physical activity is combined with dietary changes, there is greater potential for healthier outcomes and sustained habits.

Prenatal physical activity is an attractive intervention strategy for EGWG as it simultaneously promotes a multitude of maternal health benefits (e.g., reduction in risk of gestational diabetes, pre-eclampsia, depression, and body image dysphoria) [28–30]. Moreover, there is a clear need to identify effective strategies to increase prenatal physical activity as approximately 80% of pregnant women are not meeting recommended guidelines [31–33]. A combination of knowledge, skill building, motivation, and stress management may increase the likelihood of consistent engagement in regular physical activity throughout pregnancy. Studies have found that pregnant women often struggle with maintaining motivation to exercise during pregnancy due to lack of time and tiredness [30, 34]. Moreover, 10–51% of women experience mental distress and/or mood disorders [35–37] during pregnancy, exacerbating low energy and motivation to be active. Poor mood during pregnancy is also associated with compromised sleep quality [38, 39]. In turn, poor sleep quality and depressed mood may hinder the physical activity patterns that have been shown to significantly reduce depressive symptomatology and improve sleep quality during pregnancy [40, 41].

Little is known about the triadic relationship among physical activity, mood, and sleep during pregnancy and how they may collectively mitigate EGWG. Independently, studies have found that both poor sleep and mood are associated with EGWG [26, 42–45]. Additionally, women who exhibit high self-regulation have a better understanding of how physical activity benefits their mood and sleep and may be more motivated to continue with activity into the postpartum period [46, 47]. Given these inter-connected relationships, EGWG interventions may be more effective if they simultaneously target physical activity, mood, and sleep.

Social support is another factor that may mitigate adverse perinatal outcomes [48, 49]. Social support may reduce the risk of postpartum depression [49] and stress in the perinatal period [50]. Social support further enhances the likelihood of engagement in regular physical activity in the perinatal period [51]. The My Baby My Move + (MBMM +) intervention provides social support by incorporating a peer-led intervention delivery platform. Peer-led support interventions are not only cost-effective [52] but may be a more accessible and/or attractive option for pregnant women and increase the feasibility of participation. This paper presents findings related to the survey and qualitative interview data that were collected to assess the feasibility of the MBMM + intervention based on the following constructs from Bowen’s feasibility framework: acceptability, demand, implementation, and practicality [53].

## Methods

### Study design

We conducted a pilot randomized controlled trial (RCT) to test the feasibility [53] of the MBMM + online intervention compared to an online childbirth education control. The pilot RCT included two cohorts totaling 49 participants. The first cohort (Fall Cohort) participated soon after the onset of the COVID-19 pandemic from August 2020 to November 2020 and included 21 participants (intervention = 11 and control = 10). The second cohort (Spring Cohort) consisted of 28 participants (intervention = 14 and control = 14) and was conducted between April 2021 and June 2021.

### Recruitment

Pregnant women were recruited from healthcare clinics as well as through advertisements on social media (e.g., Facebook, parent groups, and community organizations) and listservs. Eligibility criteria for the first cohort included women 18–46 years of age; residing in Denver, Colorado, and surrounding areas with a singleton pregnancy between 8 and 14 weeks in gestation who were self-reportedly underactive (< 120 min of weekly moderate-intensity physical activity); willing to be randomized; and able to obtain medical clearance for physical activity during pregnancy. Given difficulties with recruiting pregnant women during the pandemic, eligibility criteria were expanded in the second cohort to a wider gestational age of 6–14 weeks and to include more active participants (< 150 min of weekly moderate-intensity physical activity). Eligibility was determined by a MBMM + study team member administering a brief screener by phone (questions included age, gestational age, physical activity, general health, and availability to participate) or an online screener through Research Electronic Data Capture electronic (REDCap), a secure, online data capture system [54].

### Procedures

Study participants’ approval was obtained from the Colorado Multiple Institutional Review Board (COMIRB #19–1366). Eligible participants provided informed electronic consent and completed an online baseline assessment (T1) using REDCap and obtained medical clearance to exercise from their healthcare providers in the form of a signed letter, which was then either delivered in paper form or scanned and emailed to the study team. Following the completion of the consent form and baseline assessment, participants were randomly assigned to either the intervention (MBMM + program) or control arm (childbirth education group) using a pre-generated table program manual for either the intervention or control.

Due to the COVID-19 pandemic, the intervention and the control curriculum were adapted from their original in-person formats to be delivered online. Instead of asking participants to meet at a recreation center to distribute materials, study team members dropped off study-related materials to each participant’s home (both intervention and control participants) including a Tanita scale and study arm-specific program manual. MBMM + intervention participants also received a yoga mat and pedometer.

### *MBMM* + *intervention*

The guiding theoretical framework for the MBMM + intervention was based on the social cognitive theory (SCT) and the social support theory [55]. These theories were selected as they include theoretical constructs such as self-efficacy, behavioral skills (e.g., self-regulation and problem-solving), and social support; all of which are strong change agents and shown to be effective in initiating and maintaining physical activity [56, 57]. Intervention activities included goal setting, which was supported by the daily tracking logs, to promote self-monitoring, which is associated with self-efficacy, and discussion around addressing barriers to regular physical activity (problem-solving). Intervention strategies and theoretical components are summarized in Table 1. These agents of behavioral change have been shown to not only increase physical activity but to sustain regular physical activity beyond the intervention. The intervention also promoted the connection between participants through live online conversations and group discussions.

**Table 1:** MBMM + MBMM+ Intervention strategies and theoretical components

Weeks of MBMM +	MBMM + intervention strategy	Social cognitive theory (SCT) and social support theory components
Baseline Participant Packet	Increase prenatal exercise knowledge, stress management, sleep hygiene and strategies, barriers, and solutions to physical activity	Behavioral capability, observational learning, expectations, self-efficacy, and reinforcements
Week 1 Sessions 1 & 2	Increased prenatal exercise knowledge and goal setting	Behavioral capability, observational learning, expectations, self-efficacy, and reinforcements
Week 2 Sessions 3 & 4	Stress reduction technique^a^, sleep strategies, awareness of values, knowledge of sleep, weight, and physical activity triad	Behavioral capability, observational learning, and reinforcements
Week 3 Sessions 5 & 6	Stress reduction technique^a^, goal revisions, increased awareness, and weight gain and stress relationship	Expectations, self-efficacy, and reinforcements
Week 4 Sessions 7 & 8	Stress reduction technique^a^, social support, goal management, benefits of yoga, and safe prenatal yoga practices	Behavioral capability, observational learning, expectations, and reinforcements
Week 5 Sessions 9 & 10	Stress reduction technique^a^, barriers, and solutions to physical activity, and identifying social and environmental supports	Behavioral capability, self-efficacy, and reinforcements
Week 6 Sessions 11 & 12	Stress reduction technique^a^, dimensions of wellbeing, prenatal nutrition, and mindful eating	Behavioral capability and reinforcements
Week 7 Sessions 13 & 14	Stress reduction techniques^a^, stress management, and gratitude	Behavioral capability, self-efficacy, and reinforcements
Week 8 Sessions 15 & 16	Stress reduction technique^a^, physical activity during postpartum, preparing for the postpartum, postpartum self-care, and commitment letter	Behavioral capability, expectations, reinforcements, and self-efficacy

^a^Stress reduction techniques included breathing exercises, mindfulness practices, and stretching

Up to 4 weeks prior to the first online MBMM + session, intervention and control participants received a starter educational packet delivered to their homes by study team members. The packets included study-arm-related material. The intervention educational packet included basic information related to exercising and managing stress during pregnancy. The MBMM + intervention consisted of 16 online group sessions (60-–75-min session, two times a week for 8 weeks). Sessions were held on weekday evenings, as these times were preferred by recruited participants. Each online intervention group session consisted of a didactic portion in which educational materials were discussed live over Zoom, followed by an experiential physical activity component (i.e., walking or prenatal yoga facilitated by a certified prenatal yoga instructor). Live online didactic components covered information such as overcoming barriers to physical activity, goal setting, self-monitoring, problem-solving, topics related to mood (stress management, family/work balance, emotional eating, and the “wellness wheel”), sleep hygiene (setting routines, avoiding stimulants, and environmental supports), and additional physical activity topics (managing physical activity in postpartum and overcoming relapses). The second half of each session included an experiential component that involved a combination of meeting with peer leaders to discuss goals and barriers, designating time for walking at a moderate intensity based on national recommendations [58], and/or live online prenatal yoga (facilitated by a trained prenatal yoga teacher).

The MBMM + intervention was delivered by peers (i.e., women from the community who were either pregnant at the time and engaging in regular physical activity or a new mother (< 2 years postpartum) who regularly engaged in physical activity during a previous pregnancy. Peer leaders were recruited from local clinics, community centers, and through social media to facilitate intervention sessions. Eligible peer leaders participated in a hiring process (i.e., interviews) and were selected based on their community ties and ability to create positive atmospheres for pregnant participants. The PI and study team conducted peer leader training sessions (1.5–2 h, dependent on questions raised by peer leaders) before the onset of the intervention to cover topics including how to engage in safe and effective physical activity during pregnancy, an overview of behavioral change through social cognitive theory, the behavioral skills targeted by the intervention, and ways to garner social support, leadership and communication skills, and sensitivity training related to mental health. Each peer leader met with a small group of participants live over Zoom (an average of four participants per group) during each MBMM + session to help participants set physical activity goals, brainstorm ways to overcome potential barriers, and provide continual encouragement and motivation. Peer leaders recorded weekly goals for each participant in the small group on an online questionnaire submitted to the study team. The study team including the PI and program coordinator regularly met with the group peer leaders to debrief sessions and address any unanticipated issues that may have been submitted through participant feedback and to make any adaptations needed. Though the meetings were held online rather than the intended in-person format, all distributed materials and guided discussions remained the same.

### Control group

The study team developed a childbirth education curriculum called “Baby Basics” for the control group to account for similar time and attention as the intervention group. A packet of online materials related to various prenatal topics included the biology of pregnancy, mitigating symptoms of pregnancy-related nausea, selecting a health care provider, and establishing a support system was distributed 4 weeks prior to the start of the 8-week online sessions. Though the control group also met online rather than the originally planned in-person format, all materials including the curriculum booklet remained the same. All control group participants were invited to attend 16 online sessions (2 × week for 8 weeks). Sessions lasted approximately 60 min and were held on weekday evenings at the same time and for the same duration as the intervention group, as these times were preferred by recruited participants. The control curriculum included evidence-based recommendations for birth planning, postpartum care, breastfeeding, and infant care from entities such as the American Academy of Pediatrics [59] and the American College of Obstetricians and Gynecologists [60]. The curriculum was reviewed by a board-certified obstetrician-gynecologist. The control group facilitator was a member of the study team and a trained Certified Lactation Counselor and Childbirth Educator who received research training in EGWG and perinatal health behaviors.

#### Assessment instruments

At baseline (T1), participants in both the intervention and control arms were asked to complete objective assessments by logging their weight for three consecutive days both pre-intervention (T1) and post-intervention (T3) via photos of the provided scales. Intervention participants were also asked to complete weekly intervention tracking logs, which consisted of daily reports of weight, physical activity, sleep, and mood patterns throughout participation in the MBMM + intervention. Tracking logs were collected weekly by the study team through email. Participants were also asked to complete a survey at T1 that included the following validated measures: the Leisure-Time Exercise Questionnaire [61] which is commonly used to measure exercise behavior in pregnant women, the Edinburgh Postnatal Depression Scale [62] which detects depressive symptoms in prenatal and postpartum women, and additional validated measures including the general anxiety disorder-7 scale (GAD-7) [63], the Perceived Stress Scale (PSS) [64], the Mindfulness Attention Awareness Scale-5 (MAAS-5) [65], and the Pittsburgh Sleep Quality Index (PSQI) [66].

A mid-point assessment (T2) was completed 6 weeks into the intervention for both intervention and control groups using REDCap and at their convenience. The T2 assessment contained a modified survey questionnaire measuring weight, as well as questions from the Godin, the EPDS, GAD-7, PSS, MAAS-5, and PSQI. Finally, participants from both the intervention and control groups completed the online survey consisting of the aforementioned validated measures and weight assessment at the end of the intervention (T3) (12 weeks post-baseline) using REDCap. Data from the weight measurements, tracking logs, and validated survey measures will be reported in subsequent manuscripts. Upon completion of the randomized controlled trial, participants had the opportunity to also participate in a survey that assessed the feasibility of the MBMM + intervention.

#### Feasibility survey

The feasibility survey was based on Bowen’s feasibility framework [53]. The constructs included *acceptability*, which assesses the reactions from the participants, *demand*, which estimates the use of the intervention, *implementation*, which focuses on the manner in which an intervention can be implemented as planned, and *practicality*, which explores the resources, time, and commitment required to successfully execute an intervention. The feasibility survey questionnaire consisted of 24 multiple choice and open-ended questions regarding different aspects of the program, including “What did you like most about The MBMM + Program?**”** (acceptability), "How helpful did you find the following program components: (i.e., electronic materials, daily tracking log, small group sessions with your peer leader)?” (implementation), and "How helpful did you find the following program components (i.e., attending program sessions, wearing the pedometer, setting goals)?” (practicality). After completion of the T1 and T2 assessment surveys, participants received a $30 electronic gift card.

Participants in both the intervention and control groups were also randomly selected to participate in semi-structured interviews at T3, in which 19 participants across both cohorts in the intervention and control groups participated. Interviews were conducted over Zoom by study team members (JL, RL, and JW) who are trained in collecting and analyzing qualitative data. Interviews included questions related to the feasibility of the intervention or control as well as the strengths and limitations of each program. For example, participants were asked, “Which part of the program did you enjoy the most, and why?” (acceptability) and “Were you able to attend the sessions with your work schedule?” (practicality). Interviews lasted approximately 25 min (12–37 min). After completion of each interview, participants received a $30 electronic gift card.

#### Peer leader assessments

Throughout the delivery of the intervention, the study team met weekly with the lead peer leader to debrief and address any arising issues. In addition to the weekly debriefing meetings, each peer leader completed session feedback forms that were monitored by the study team to assess the quality control and feasibility of the intervention. Peer leaders noted the group’s progress on meeting weekly goals, overall discussion related to identified barriers, recommended evidence-based strategies, and recommended action steps in weekly feedback forms. The study team reviewed these forms following each session and monitored the intervention’s delivery to inform program implementation. Peer leaders also completed a brief role-related survey at T3. This survey assessed their likelihood of returning as a peer leader in the future, the impact and usefulness of the programmatic components (e.g., tracking logs, small group sessions, and didactic presentations) as well as their preparedness in effectively building relationships with the participants (e.g., training and study team support).

#### Assessing feasibility

Feasibility was assessed using a combination of surveys, interviews, and programmatic records. Descriptive statistics from continuous and categorical survey data were computed using SPSS software [67]. Qualitative interviews were audio-recorded, transcribed verbatim, and stored on a password-protected computer. Transcripts were analyzed by members of the study team (RL, JW, and JL) using a deductive approach in ATLAS.ti [68] software based on constructs from Bowen’s feasibility framework [53]. Programmatic records were reviewed including attendance tracking, intervention tracking log completion, and peer leader feedback forms to inform findings related to demand and implementation of the MBMM + program.

## Results

### Demographics

Demographic data for the full sample can be found in Table 2. The average age of participants was 31.8 years, and the median age was 32. 64.3% of the total sample had no other children at home and 4.7% had 1 or more children. Approximately 22% of participants identified as Hispanic or Latino, 81.6% Caucasian, and 8.2% African American. Approximately 14.3% reported a household income of less than $49,999, 18.4% reported $50,000–$74,999, 65.3% reported $75,000 or more. The sample was highly educated with approximately 81% a recipient of a college degree or more and 18.4% more than a college degree. Most participants (81.6%) had full health care coverage, only 8.4% had partial coverage or Medicaid (federally and/or state-funded health care). The majority of the sample were married (81.6%), while 18.4% were single or partnered. Over half of the participants (53.1%) had a BMI placing them in the “normal weight” category, while 8.2% were underweight, 20.4% overweight, and 18.3% obese.

**Table 2:** Demographics of full sample (*n* = 49)

Demographics	Total (***n*** = 49) ***N*** (%)	Interventio***n*** (***n*** = 25)	Control (***n*** = 24)
Age (years)			
Mean	31.8	32.4	31.2
Median	32	31	31
SD	4.6	5.7	3.0
Number of children in household			
0	32 (65.3)	13 (52.0)	19 (79.2)
1 or more	17 (34.7)	12 (48.0)	5 (20.8)
Ethnicity			
Hispanic or Latino	11 (22.4)	7 (28.0)	4 (16.6)
Non-Hispanic or Latino	38 (77.6)	18 (72.0)	20 (83.3)
Race (could select more than one)			
Caucasian	40 (81.6)	20 (80.0)	20 (83.3)
African American	4 (8.2)	2 (8.0)	2 (8.3)
American Indian, Asian or Pacific Islander or other	9 (18.4)	4 (16.0)	3 (12.5)
2020 household income			
≤ $49,999	7 (14.3)	3 (12.0)	4 (16.6)
$50,000–$74,999	9 (18.4)	5 (20.0)	4(16.6)
≥ $75,000	32 (65.3)	17 (68.0)	15 (62.5)
Prefer not to answer	1 (2.0)	0	1 (4.2)
Education level			
Less than a college degree	9 (18.4)	6 (24.0)	3 (12.5)
College degree or more	40 (81.6)	19 (76.0)	21 (87.5)
Health insurance coverage			
Full coverage	40 (81.6)	19 (76.0)	21 (87.5)
Partial coverage or Medicaid (federally and/or state-funded healthcare	9 (18.4)	6 (24.0)	3 (12.5)
Marital status			
Single or partnered	9 (18.4)	6 (24.0)	3 (12.5)
Married	40 (81.6)	19 (76.0)	21 (87.5)
BMI category			
Underweight (< 18.5)	4 (8.2)	2 (8.0)	2 (8.3)
Normal weight (18.5–24.9)	26 (53.1)	11 (44.0)	15 (62.5)
Overweight (25.0–29.9)	10 (20.4)	7 (28.0)	3 (12.5)
Obese (≥ 30.0)	9 (18.4)	5 (20.0)	4 (16.6)

#### Feasibility survey and qualitative interview findings

A total of 14 MBMM + participants completed the feasibility survey, and 15 intervention and 4 control participants completed interviews at T3. Table 3 displays a detailed description of the survey and interview feasibility results.

**Table 3:** Feasibility of MBMM + across cohorts one and two

**Feasibility constructs**	**Feasibility outcomes**	**Supporting data** (**participant description if qualitative)**
**Acceptability**	Satisfaction and enjoyment	100% (*n* = 13) *somewhat* or *very* satisfied; 5-item Likert scale (1 = not at all to 5 = very satisfied) -C2 participants
		69% (*n* = 9) *very much* or *much* enjoyed*,* 31% (*n* = 3) *enjoyed;* 5-item Likert scale (1 = very not much to 5 = very much) -C2 participants
		4.6/5 overall enjoyment rating of being a peer leader -C2 peer leaders
		"I really enjoyed it. I think it was a great experience. It was informative, and honestly, probably the best part of it was the accountability." -C2 VM, overweight
		"I liked having time on the calendar to speak with other women experiencing the same things. The pointed questions from the peer leader each session made the discussions more valuable than just chatting with a pregnant friend." -C2, FTM, overweight
		"[I enjoyed] The support provided by the ladies and peer leader in my breakout group. It was nice to be able to talk about real concerns, get advice from second time moms and feel supported." -C2, FTM, overweight
		"This has been such a wonderful experience. I was hired to be the lead peer leader but I learned so much from these women too! Thank you for your support and confidence throughout the program" -C2 lead peer leader
	Intent to continue use	100% of peer leaders identified facilitating as a learned skill to continue using -C2 peer leaders
		"I think that whole piece of establishing like smart goals and realistic goals helped me [in the] postpartum." -C1, FTM, normal weight
		"I think I'm going to use [relaxation techniques] even after pregnancy because I do have a lot of children, so I tend to get stressed out easily". -C2, VM, normal weight
		"I still text our smaller group from time to time and follow-on Instagram! Such a great group of women. Very appreciative for this experience" -C2, peer leader
	Perceived appropriateness	"I just think that it's just a really great program to start. It doesn't matter if it's your first baby, your second, your third, your 17th, or whatever, I think that it's a great way to touch on different topics that you're not really thinking about". -C1, VM, obese
		"I think for the purposes of a wide ability in terms of the group, [the yoga] was appropriate." -C2, VM, overweight
**Demand**	Actual use	5.32/8 average tracking log completion -C1 & C2 participants
		"I think the [tracking] log was probably the most helpful thing just for objectively looking at how much exercise I was doing". -C2, FTM, overweight
		"I found it really beneficial to have to weigh myself every morning." -C2, FTM, overweight
		"Everything was really informative. I really enjoyed the accountability of the small groups." -C2, FTM, overweight
		"The yoga mat was really helpful. I use that a lot during the sessions and also [in postpartum]". -C1, FTM, underweight
		"I read the [manual], I did have the digital version at first, and on my phone, I can see it and I can move it around, especially when I opened it up on my iPad. It gave me the ability to follow along" -C1, VM, obese
		"It was just good to see [the tracking log] as a reminder that I was on track" -C2, FTM, normal weight
		"Having the information on the food, like what foods to eat, what foods to stay away from, and having the recipes on there, I was able to make one of them, one of the chicken ones and, and so that's really good to include that information" -C1, FTM, underweight
	Perceived demand	92% (*n* = 12) *likely* or *extremely likely*, 8% (*n* = 1) unlikely to recommend; 5-item Likert scale (1 = unlikely to 5 = extremely likely) -C2 participants
		17/25 (68%) of intervention participants completed 10 or more session (i.e., compliant); 16 total sessions -C1 & C2 participants
		100% of peer leaders would recommend MBMM + to pregnant women -C1 & C2 peer leaders
**Practicality**	Quality of implementation	100% completion of peer leader session feedback forms -C1 & C2 peer leaders
		100% attendance of program sessions by a study team member
		“So when we went through [the workbook] together on the calls, it made that resource much more valuable because I was actually using it” -C2, FTM, obese
	Positive/negative effects on target participants	The #1 identified barrier to being a peer leader in the future was time constraints
		"We touched base on [stress management] in the class and I did learn some techniques to [reduce] this….And honestly, I really, really, really think that that is the one of the biggest reasons hy I'm probably not on [anxiety and depression] medication right now" -C1, VM, obese
		"Our group developed such a great, comfortable, supportive relationship that was so exciting to watch develop throughout the program." -C2, peer leader
		"They [participants] loved the yoga! There was also a discussion about how much they appreciated that the curriculum included postpartum [material]. A few women mentioned that this was not already on their mind and that they were so happy we brought it up to think about and plan for their self-care once baby arrives. Postpartum depression acknowledgement was also appreciated" -C2, peer leader
		"I love the body scan activity [relaxation technique] and hope the women find these activities helpful. I have been incorporating them back into my life" -C2 peer leader
	Ability of participants to carry out intervention activities	"If the whole thing was in person, and we still tried to meet twice a week, I think that it would decrease participation just because of logistics, like needing a babysitter for the other kids. But a hybrid of having a couple in-person options could be cool. -C2, FTM, overweight
		"We were just in Las Vegas walking, making sure that I'm still walking like 15 min a day at least" -C2, VM, normal weight
		"I still use [relaxation techniques] today. I still sit on the floor, I use my yoga mat. And I still stretch." -C1, VM, obese
		"I think, [the program] is definitely applicable for all different types of people and levels [of fitness/knowledge] and all of it and so I absolutely would recommend it to anybody." -C2, FTM, overweight
		"Our group chatted quite a bit, sometimes we needed more time, but we were able to stay on. When safe, the in-person walks would be so wonderful! Our group spoke about how we would really all like to do this" -C2, peer leader
**Implementation**	Success or failure of execution	85% average attendance rate of compliant intervention (*n* = 17), 68% average attendance rate of intervention group (*n* = 25) -C1 & C2 participants
		“And so I think the way to that it's structured, I think, is definitely applicable for all different types of people and [physical ability] levels" -C2, FMT, overweight
		“Facilitation went well, was happy to receive advice from the study team during my breakout. I feel like the guide for today's session perfectly outlined what we needed to go over in the amount of time we had.” -C2, peer leader
		"I think creating an environment where they didn't feel that they were being reprimanded if they didn't complete a goal, and that we could all talk through goals together helped" -C2 peer leader
	Degree of execution	All peer leaders found it *"very helpful"* for a study team member to attend sessions -C1 and C2 peer leaders
		"[The study team] meeting me at work at the beginning, and then she dropped stuff off at my house. And I think that was probably the most helpful thing. Just having the supplies coming to me and not having to go drive anywhere." -C1, FTM, normal weight
	Amount and type of resources needed to implement	"Having a scale and a yoga mat, those were really huge perks of the program" -C2 FTM, overweight
	Factors affecting implementation ease or difficulty	"The gift cards help. I mean, I know, it's not a lot, but I think if there's some incentive, it always helps." -C1, FTM, normal weight
		"I would say maybe an early afternoon session would work for my schedule, I mean, I was working part time in the evenings and then I'm trying to just get my household aligned and try to participate in the group and it was a little difficult" -C1, FTM, obese
		“[The session was] really good, it was awesome getting the email from the study team reminding us of today's meeting flow.” -C2, peer leader
		“I appreciate having the option to chat [with the study team] if we had a question.” -C2, peer leader

*C1* Cohort 1, *C2* Cohort 2, *FTM* First time mom, *VM* Veteran mom

Data analysis suggested high acceptability; 100% of participants stated they would recommend the program to another pregnant individual and all respondents were “somewhat satisfied” or “very satisfied” with the program. Participants appreciated how intervention materials were delivered in multiple formats (i.e., hard copies and electronically). Among peer leaders, the average score of enjoyment related to the facilitation of the group sessions was 4.6 (1 (not at all enjoyable) to 5 (highly enjoyable)). All peer leaders found it “very helpful” to have a member of the study team attend each session. The control intervention was also deemed to be acceptable; over 85% of control group participants were satisfied with the program and another 72% of participants were “likely” or “extremely likely” to recommend the control program to a friend.

Demand was measured by attendance and participation in the MBMM + intervention sessions. Approximately 2/3 (68%) of intervention tracking logs were completed throughout the intervention, leading to 68% of participants being categorized as “compliant” (attended 10 or more sessions) with the average compliant participant attending 85% of sessions. While the final sample was small, there were many women who showed interest in the intervention and were not eligible. Interested women that were outside the gestational age parameters, were physically active for more than 150 min per week, or did not receive medical clearance from their health care provider were not permitted to participate.

Implementation was measured by the ability of participants and peer leaders to implement all intervention activities. Participants qualitatively shared how the intervention materials (yoga mat, tracking logs, and manuals) enhanced their experience in the program and enabled them to participate in the weekly didactic and experiential portions of the program. Participants also mentioned how the incentives (diaper raffles and gift cards) were helpful tools to increase accountability and attendance. One participant expressed appreciation for the delivery of materials: “I was at home with my other kiddos, so it was nice to have the materials brought to me”. Additionally, all peer leaders completed 16/16 session feedback forms which suggest implementation success related to this component of the MBMM + program. The program coordinator was present for all intervention group meetings, and the PI attended approximately half of the MBMM + sessions and at least two sessions for each cohort for the control group to assess the quality of program delivery and implementation.

Practicality was assessed by the ease with which participants could realistically participate in terms of time, space, and materials. Participants gave positive feedback regarding the timing of the sessions. For example, a fall cohort intervention participant stated: “I did like the sessions later in the day, just because they fit with my work schedule”. Additionally, the online format enabled participation, as one member of the fall cohort explained: “I wouldn’t have been able to be part of the group if it wasn’t on Zoom”. Those with older children expressed that the flexibility of the remote model made it easier to attend because they did not have to find childcare.

#### Peer leader component

Peer leaders reported a pre-post change in prenatal exercise and weight gain knowledge following the training with the study team. In addition to increased knowledge from the trainings, peer leaders enjoyed participating in the MBMM + intervention; 100% of peer leaders stated they would be peer leaders in the future and would recommend this program to pregnant women in their communities, as either a participant or a leader.

## Discussion

Our findings suggest that the MBMM + intervention is feasible. High acceptability was reported by both participants and peer leaders, suggesting that community-based intervention delivery through peer mentors may be an effective strategy in translating prenatal physical activity programs into a community setting [69–73]. Extant studies indicate peer-delivered interventions (i.e., trained volunteer peers) may be as effective as clinical staff-delivered interventions in helping women improve and maintain physical activity [71, 72]. Pregnant women have reported valuing interpersonal interactions with other pregnant women (i.e., peers they can identify with) to motivate and support behavior change such as increased physical activity [71, 73, 74].

Similar to prior research findings [75–77], our MBMM + participants conveyed that peer leaders provided a sense of support and guidance for participants. A recent study measuring perceived social support during pregnancy and early childhood also found positive effects of peer support on improving mood and reducing anxiety, stress, and isolation during pregnancy [78]. This was particularly true for primiparous participants in our study, who appreciated the peer leaders and veteran mother’s lived experiences of pregnancy and the postpartum period. In addition to the social and motivational support they offer, peer leaders from the community may be key in encouraging and translating prenatal health behaviors recommended by healthcare providers such as weight, exercise, sleep and stress management guidelines, and referral follow-ups.

The MBMM + intervention also demonstrated high demand among pregnant women due to high attendance and completion of assessments. Many existing EGWG interventions focus on testing clinical interventions in healthcare settings only [42, 79–81]. However, a recent systematic overview found that women who participated in group prenatal care (provider- and facilitator-led) had improved attendance rates of prenatal visits [82]. Additionally, utilizing peer leaders may mitigate reliance upon study team members and provide a low-cost opportunity for dissemination [71, 72] of evidence-based practices, improving the likelihood of future scalability. Interventions such as MBMM + that utilize a peer-led approach may translate to increased accessibility of prenatal care interventions that support the adoption of positive health behaviors and mitigate EGWG.

Implementation of the MBMM + was successful in this pilot study. The intervention was originally developed to be delivered in-person at community recreation centers but was adapted for online delivery due to the COVID pandemic. A recent study comparing in-person and online health coaching that targeted physical activity, weight change, and healthy eating demonstrated no significant differences between group formats for physical activity and weight change outcomes [83]. With an increasing demand for virtual programming following the COVID pandemic, a delivery modality of both online and in-person sessions (hybrid) may best support expectant parents while also increasing social support during pregnancy and the postpartum period [84, 85].

Additionally, the idea of a hybrid format translated into high practicality related to participation in the MBMM + intervention. While participants expressed that in-person sessions may have fostered more social support, they also acknowledged the convenience of online sessions, eliminating potential barriers to in-person participation (e.g., transportation, transportation costs, and cost of childcare), which have been cited as barriers to prenatal physical activity even before the pandemic [30].

Findings from this feasibility study suggest that the MBMM + intervention is feasible and also highlight ways to enhance the future refinement of MBMM + . Overall, participants reported that they enjoyed the small group discussions with their peer leaders. Some participants voiced a need for having even more time for a small group discussion in future programming. Additionally, participants suggested that they really enjoyed the stress management activities and desired more examples to be shared. A few participants suggested varying times when sessions were held would be helpful, particularly with some sessions being offered in the afternoons. Lastly, many participants conveyed a desire to meet again in the postpartum period.

### Limitations

There are several limitations to this feasibility study. First, due to the COVID-19 pandemic, the intended curriculum was created for in-person facilitation, which had to be adapted for the safety of participants and study team members, causing the need for the program curriculum to be adjusted to an online platform and virtual sessions via Zoom. This online programming changed the delivery of the experiential component (e.g., in-person walking and yoga sessions). In addition to the restructuring of the program, the recruitment of pregnant women presented a challenge. During the first cohort (Fall of 2020), COVID-19 vaccines were not yet available, and many pregnant women were unable to see their OBGYNS as early as recommended in their pregnancy for safety concerns, thus limiting patient-clinic relations. The limited access to clinics/providers hindered the ability to obtain medical clearance forms. Recruitment issues resulted in a small, relatively homogeneous sample size.

## Conclusion

In summary, these findings demonstrate that MBMM + is a feasible peer-led intervention with a high degree of acceptance, demand, implementation, and practicality. Future research that tests a hybrid delivery of MBMM + with a larger sample size is needed. Findings from this feasibility study will inform the future refinement of My Baby My Move + to be tested in a larger, well-powered RCT, as well as may inform the future development of other perinatal behavioral interventions that include peer-based facilitation.

## Data Availability

The datasets generated and/or analyzed during the current study are not publicly available due to sensitive data and sharing agreements but are available from the corresponding author upon reasonable request.

## Abbreviations

MBMM +: My Baby My Move +
GWG: Gestational weight gain
EGWG: Excessive gestational weight gain
